# Communicative knowledge pervasively influences sensorimotor computations

**DOI:** 10.1038/s41598-017-04442-w

**Published:** 2017-06-27

**Authors:** Anke Murillo Oosterwijk, Miriam de Boer, Arjen Stolk, Frank Hartmann, Ivan Toni, Lennart Verhagen

**Affiliations:** 1 Radboud University, Donders Institute for Brain, Cognition and Behaviour, Nijmegen, The Netherlands; 2 Erasmus University Rotterdam, Rotterdam School of Management, Accounting and Control, Rotterdam, The Netherlands; 3 Helen Wills Neuroscience Institute, University of California, Berkeley, USA; 4 University of Oxford, Department of Experimental Psychology, Oxford, United Kingdom

## Abstract

Referential pointing is a characteristically human behavior, which involves moving a finger through space to direct an addressee towards a desired mental state. Planning this type of action requires an interface between sensorimotor and conceptual abilities. A simple interface could supplement spatially-guided motor routines with communicative-ostensive cues. For instance, a pointing finger held still for an extended period of time could aid the addressee’s understanding, without altering the movement’s trajectory. A more complex interface would entail communicative knowledge penetrating the sensorimotor system and directly affecting pointing trajectories. We compare these two possibilities using motion analyses of referential pointing during multi-agent interactions. We observed that communicators produced ostensive cues that were sensitive to the communicative context. Crucially, we also observed pervasive adaptations to the pointing trajectories: they were tailored to the communicative context and to partner-specific information. These findings indicate that human referential pointing is planned and controlled on the basis of partner-specific knowledge, over and above the tagging of motor routines with ostensive cues.

## Introduction

Referential pointing actions can convey complex communicative intentions by means of motorically simple movements. Pointing directs the attention of an addressee to a remote referent by means of a proximate sign^1^, as when a diner points to an empty glass to request a new bottle of wine from the waiter. Planning such ‘mind-oriented’ actions, i.e. actions that are designed to change the mental state of an addressee by virtue of being recognized as communicative^2^, requires at least two systems. On the one hand, it requires a communicative system that specifies a behavioral vehicle suitable to convey the intended meaning to an addressee^3, 4^. This system relies, even for simple pointing, on conceptual information about the referential goal and the communicatively relevant characteristics of the addressee. Importantly, this information is not readily available in the environment, such as the knowledge that wine can be ordered from waiters, but not from cleaners^5, 6^. On the other hand, a sensorimotor system needs to guide the finger towards the referent object, similarly to ‘object-oriented’ actions that are designed to instrumentally change the physical environment^7–9^. Here we study the interface between the communicative and the sensorimotor systems by contrasting mind-oriented and object-oriented pointing movements. These actions are experimentally tractable instances of the broader debate on the nature of the interface between conceptual knowledge and sensorimotor processes in communicative action planning^10–15^.

A number of studies have investigated the extent to which different actions, including pointing movements, are affected by a communicative intention^16–23^. The majority of the empirical evidence suggests that in interactive settings communicators flexibly adapt their actions based on communicative demands and partner-specific knowledge^5, 10, 17, 18, 21, 23–29^. However, these observations raise two questions. Firstly, do these adaptations reflect general adjustments to social context (e.g. joint vs. solo actions^30^), social setting (e.g. left vs. right addressee^18^) and social relevance (e.g. eyes open vs. closed^18, 22^), or are they specifically tailored to the current triadic interaction elicited by the communicator, addressee, and referent^23, 25^? Secondly, *how* do such communicative adaptations interface with sensorimotor computations? To answer these questions, in the current study we aim to characterize the temporal and functional nature of the interface between these computational systems. We do this by quantifying the extent to which sensorimotor processes controlling movement kinematics are malleable to the triadic relationship between communicator, addressee and referent.

Human communicative and sensorimotor systems could operate in a modular fashion, in relative isolation from each other^31, 32^, or be pervasively integrated, allowing the systems to access each other’s internal computations^33, 34^. In a ‘modular framework’, where communicative knowledge is largely encapsulated from sensorimotor computations, pointing movements could be assembled from spatially-guided sensorimotor routines and ostensive communicative cues^35, 36^. These cues draw the attention of the addressee to the communicative status of the action because she recognizes the cues as instrumentally dysfunctional^37–39^, such as emphatically holding a finger in mid-air near the empty glass when ordering the new bottle. In favor of this view, recent studies have shown how increased communicative demand promotes a slower pointing movement with a prolonged hold time^21, 22^. However, it is also possible that conceptual information about the referential goal, the characteristics of the addressee, and the communicative context change the spatially-guided sensorimotor routines, i.e. the computations of the control parameters that lead to a particular pointing trajectory^34, 40–42^. Such a ‘pervasive integration framework’ is supported by recent evidence showing that the trajectories of instrumental actions are influenced by the social context and specifics of the addressee, including their task-relevance, location, and gaze^43–46^. These findings raise the question whether such adaptations are driven by stimulus factors akin to how object, distractor, and spatial context features guide goal-directed actions, or by conceptual knowledge that is part of the communicative intention and independent of sensory evidence.

**Figure 1:**
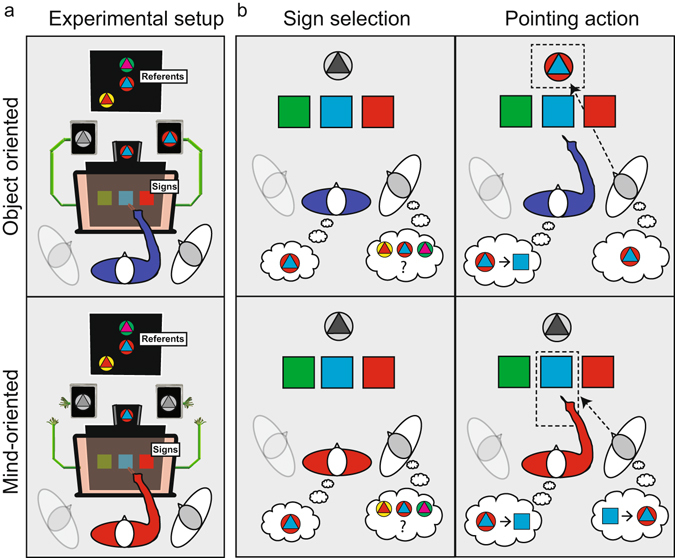
Experimental task. A schematic illustration of the experimental setup (**a**) and the trial interactions (**b**) in the object-oriented (top row) and mind-oriented (bottom row) action conditions. In this example the communicator plays the game with the right addressee, the left participant is the onlooker. Participants were asked to jointly open a vault. Only the addressee could enter the code (one of the referents), but only the communicator knew the correct code. To solve the game, the communicator needed to signal the correct referent to the addressee by pointing to one of the three signs, moving through a position-detection system. (**a**) The participants were informed that the vault’s computer system would analyse online the communicator’s pointing movement towards the signs and display the corresponding referent to the addressee (object-oriented trials). However, sometimes the display would not work (mind-oriented trials). On those trials, the addressee could infer the corresponding referent by means of the pointing movement of the communicator. (**b**) Left panels depict the sign selection phase (identical for object- and mind-oriented conditions) in which the communicator selects the sign that denotes the correct referent most clearly (the duration of this phase is the ‘reaction time communicator’). Right panels depict the pointing action towards the chosen sign. Subsequently, the addressee is either visually informed about the identity of the computer matched referent (object-oriented) after the movement had been completed, or infers the correct referent from the pointing movement itself (mind-oriented).

In this study, we investigate the plausibility of the modular and pervasive interaction accounts of how communicative actions are planned. To this end we have developed a communicatively interactive game that involved a communicator, an addressee, and an onlooker, all centered around a communicative medium with signs and referents (Fig. 1). The study is focused on the mental processes of the communicator, as derived from analysis of the durations, the end-point locations, and the spatio-temporal dynamics of the communicator’s pointing movements. The experimental design manipulated three features of the triadic relationship between communicator, addressee, and referent that characterizes referential communicative actions^47^. First, we manipulated the action intention, contrasting referential pointing movements that conveyed information about the referent (‘mind-oriented movements’) against instrumental pointing movements that triggered the visual presentation of the referent (analogous to a button press, ‘object-oriented movements’). Importantly, we keep the visual cues, social cues and communicative context of the task stable (in all conditions the objective of the communicator was to transfer information to the addressee). Second, we manipulated the position of the addressee, by alternating the roles of the two participants on either side of the communicator (left and right) between addressee and onlooker. Third, we manipulated the capacity of three signs (located on the left, middle and right) to disambiguate between the available referents by exploiting well-established pragmatic inferences^48^. By isolating behavioral markers of the referential pointing movements evoked in this dedicated task we assess how communicative knowledge influences movement execution.

## Results

Thirteen triplets of participants (one communicator flanked on either side by an addressee and an onlooker) were asked to jointly open a vault. The vault-code, a sequence of multi-colored tokens (‘referents’), could be entered only by the addressee. However, the identity of each correct referent could be seen only by the communicator. The communicator could signal that knowledge to the addressee by pointing to one out of three single-colored tokens (‘signs’). The vault story was designed to clearly differentiate the instrumental nature of the object-oriented pointing movements from the communicative nature of the mind-oriented pointing movements (Fig. 1). Namely, in the object-oriented condition, the communicator’s pointing movement towards the signs was analyzed online by the vault’s computer system and the corresponding referent was automatically displayed to the addressee on a dedicated screen. In these trials, the addressee had to confirm this referent by selecting the identical token from the set of possibilities. In the mind-oriented condition, a broken wire prevented the vault’s computer system to display the referent to the addressee. In these trials, the addressee needed to infer the correct referent from the pointing movement of the communicator towards one of the signs. As such, the vault story used task-contingencies, rather than verbal instructions, to inform the participants of the instrumental demands of object-oriented pointing and of the communicative demands of mind-oriented pointing.

In the following sections, we report the effects of the three experimental manipulations [*action* (*object-oriented*, *mind-oriented*), *addressee* (*left*, *right*), and *sign* (*left*, *middle*, *right*)] on the duration of different phases of the pointing movements, the end-point locations of those movements, and the spatio-temporal dynamics of their trajectories.

### Movement phases and parameters

**Figure 2:**
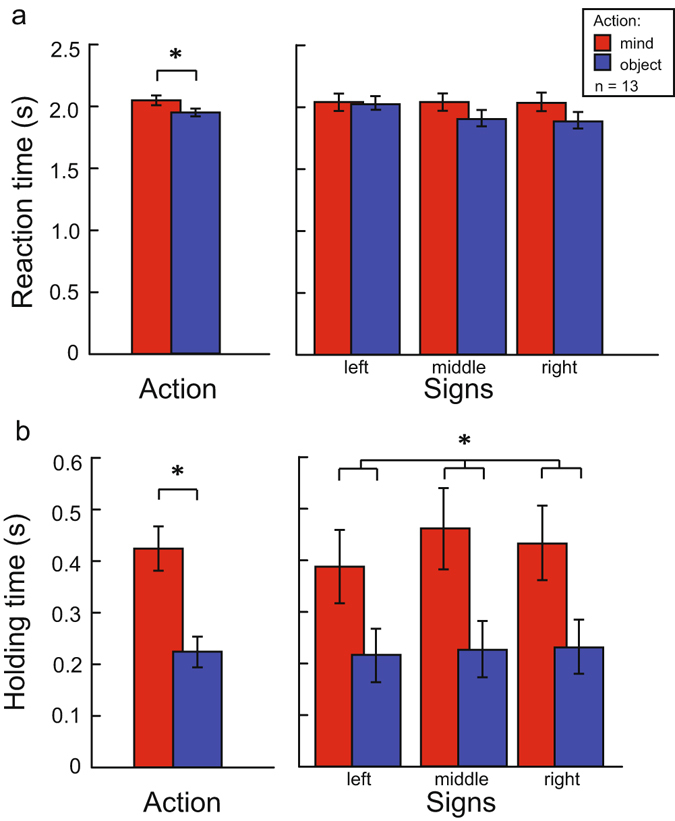
Reaction and holding times. Mind-oriented actions are represented by red bars and object-oriented actions by blue bars. (**a**) Reaction time communicator: the time from stimulus presentation to the communicator initiating the pointing movement. *Indicates a significant main effect of *action* (*p* < 0.05). (**b**) Holding time: the time that the finger was held still within the proximity of the selected sign. *Indicates significant *action* main and *action x sign* interaction effects (*p* < 0.05). Error bars represent standard errors of the mean.

There were several differences between the pointing movements evoked during mind-oriented and object-oriented trials. First, the reaction time of the communicator (the time before movement onset) was longer in the mind-oriented condition than in the object-oriented condition (2043 vs. 1945 ms; *F*(1,12) = 12.14, *p* = 0.005, ${{\rm{\eta }}}_{p}^{2}$ = 0.503; Fig. 2a; for further descriptive statistics see Table 1 and S1, available in the Supplemental material, for further statistical information see Table 2). The forward movement time (the time the finger was transported towards the sign) did not significantly differ between conditions. However, the holding time (the time the communicator’s finger was held still near the sign) was almost twice as long in the mind-oriented condition than in the object-oriented condition (426 vs. 225 ms; *F*(1,12) = 16.49, *p* = 0.002, ${{\rm{\eta }}}_{p}^{2}$ = 0.579; Fig. 2b). This effect was strongest for the sign in the middle (left sign: 387 vs. 216 ms, middle sign: 460 vs. 227 ms, right sign: 432 vs. 231 ms; *F*(2,24) = 3.77, *p* = 0.038, ${{\rm{\eta }}}_{p}^{2}$ = 0.239; Fig. 2b). The middle sign was most difficult to point out given that it was flanked by the two others. Lastly, the backward movement time (the time the hand was transported back to the starting position) was longer in the mind-oriented than in the object-oriented condition (749 vs. 704 ms; *F*(1,12) = 12.89, *p* = 0.004, ${{\rm{\eta }}}_{p}^{2}$ = 0.518). Critically, these main effects of the action’s communicative intent were not influenced by the position of the addressee. This observation suggests that modifications in the timing of the movements were used as ostensive cues, drawing attention to the communicative status and difficulty of the action but not necessarily tailoring the communicative message to the addressee.

**Table 1:** Descriptive statistics: mean and standard error of the mean for all conditions.

Action		Mind-oriented						Object-oriented					
Addressee		Left			Right			Left			Right		
Sign		L	M	R	L	M	R	L	M	R	L	M	R
RTc	*ms*	2076 (108)	2047 (103)	2061 (115)	2023 (115)	2037 (96)	2015 (115)	2057 (106)	1915 (95)	1916 (105)	2009 (84)	1909 (91)	1866 (98)
MTf	*ms*	908 (39)	900 (32)	885 (33)	875 (25)	870 (29)	871 (27)	855 (39)	876 (37)	836 (41)	864 (41)	845 (35)	860 (41)
HT	*ms*	362 (94)	458 (114)	415 (95)	412 (107)	461 (110)	449 (112)	225 (77)	249 (82)	255 (83)	207 (72)	205 (72)	207 (68)
MTb	*ms*	755 (37)	763 (33)	736 (34)	764 (26)	751 (34)	728 (32)	724 (31)	711 (30)	701 (36)	709 (26)	693 (29)	687 (28)
MTa	*ms*	1413 (38)	1410 (60)	1333 (46)	1383 (80)	1349 (77)	1269 (82)	1450 (65)	1379 (59)	1395 (60)	1367 (74)	1283 (63)	1291 (76)
TL	*cm*	39.7 (1.3)	41.6 (1.3)	44.3 (1.4)	39.2 (1.4)	41.3 (1.3)	44.3 (1.1)	40.9 (1.4)	41.7 (1.3)	44.4 (1.5)	40.0 (1.3)	41.8 (1.2)	44.7 (1.2)
PV	*cm*/*s*	100.3 (6.3)	103.0 (5.1)	102.1 (4.1)	100.4 (7.3)	101.3 (5.1)	103.5 (4.8)	102.2 (5.9)	103.7 (6.6)	107.7 (6.)	103.3 (7.3)	104.0 (7.3)	103.9 (5.6)
rtPV	%	16.9 (2.5)	13.0 (1.8)	17.8 (2.0)	15.8 (2.2)	16.2 (1.8)	18.8 (2.1)	16.7 (2.7)	15.5 (2.0)	18.8 (2.3)	16.8 (2.8)	16.2 (2.5)	21.9 (2.3)
EPx	*mm*	17.8 (1.8)	62.5 (0.9)	110.4 (1.3)	19.1 (2.0)	64.4 (0.9)	112.1 (1.5)	20.0 (1.7)	63.0 (1.0)	107.8 (0.7)	20.4 (1.7)	63.7 (0.8)	108.2 (1.0)
EPy	*mm*	15.4 (6.8)	33.1 (7.4)	52.9 (6.5)	12.5 (6.7)	35.4 (6.5)	52.3 (6.4)	19.4 (7.6)	37.1 (7.7)	52.7 (7.2)	17.4 (7.1)	37.7 (6.3)	52.3 (6.6)
EPz	*mm*	23.5 (3.5)	30.5 (3.0)	35.6 (2.7)	23.4 (3.1)	31.7 (2.7)	34.7 (2.4)	27.3 (3.6)	34.4 (3.1)	37.1 (2.8)	25.5 (3.5)	34.1 (3.1)	37.4 (3.0)

The left, middle and right signs are abbreviated with L, M and R, respectively. RTc = reaction time communicator. MTf = forward movement time. HT = holding time. MTb = backward movement time. MTa = movement time addressee. TL = trajectory length forward movement. PV = peak velocity. rtPV = relative time to peak velocity. EPx = lateral end-point, horizontal x-axis. EPy = depth end-point, forward y-axis. EPz = altitude end-point, height z-axis.

**Table 2:** Statistics: the p-value, F-value and effect size of the observed results.

	Action (Ac)	Addressee (Ad)	Sign (Si)	Ac × Ad	Ac × Si	Ad × Si	Ac × Ad × Si
RTc	0.005* (12.14) ${{\rm{\eta }}}_{p}^{2}$: 0.503	0.179 (2.00)	0.105 (2.49)	(F < 1)	0.072 (2.93) ${{\rm{\eta }}}_{p}^{2}$: 0.196	0.277 (1.35)	(F < 1)
MTf	0.085 (3.61)${{\rm{\eta }}}_{p}^{2}$: 0.231	0.197 (1.86)	0.322 (1.19)	0.107 (3.03)	(F < 1)	0.051 (3.37) ${{\rm{\eta }}}_{p}^{2}$: 0.220	0.206 (1.69)
HT	0.002* (16.49) ${{\rm{\eta }}}_{p}^{2}$: 0.579	(F < 1)	0.006* (6.28) ${{\rm{\eta }}}_{p}^{2}$: 0.343	0.088 (3.46) ${{\rm{\eta }}}_{p}^{2}$: 0.224	0.038* (3.77) ${{\rm{\eta }}}_{p}^{2}$: 0.239	0.123 (2.29)	(F < 1)
MTb	0.004* (12.89) ${{\rm{\eta }}}_{p}^{2}$: 0.518	0.138 (2.53)	< 0.001* (14.21) ${{\rm{\eta }}}_{p}^{2}$: 0.809	0.322 (1.02)	0.229 (1.57)	0.368 (1.04)	(F < 1)
MTa	(F < 1)	(F < 1)	< 0.001* (17.19) ${{\rm{\eta }}}_{p}^{2}$: 0.589	(F < 1)	0.078 (2.84) ${{\rm{\eta }}}_{p}^{2}$: 0.191	(F < 1)	(F < 1)
TL	(F < 1)	(F < 1)	< 0.001* (99.14) ${{\rm{\eta }}}_{p}^{2}$: 0.892	(F < 1)	(F < 1)	(F < 1)	(F < 1)
PV	(F < 1)	(F < 1)	(F < 1)	(F < 1)	(F < 1)	(F < 1)	(F < 1)
rtPV	0.114 (1.91)	0.008* (9.98)${{\rm{\eta }}}_{p}^{2}$: 0.454	0.001* (10.02) ${{\rm{\eta }}}_{p}^{2}$: 0.455	(F < 1)	(F < 1)	0.168 (1.92)	(F < 1)
EPx	0.150 (2.36)	0.002* (14.82) ${{\rm{\eta }}}_{p}^{2}$: 0.553	< 0.001* (1629.66) ${{\rm{\eta }}}_{p}^{2}$: 0.993	0.024* (6.69) ${{\rm{\eta }}}_{p}^{2}$: 0.358	0.001* (10.29) ${{\rm{\eta }}}_{p}^{2}$: 0.462	(F < 1)	(F < 1)
EPy	(F < 1)	(F < 1)	< 0.001* (237.49)${{\rm{\eta }}}_{p}^{2}$: 0.952	(F < 1)	0.112 (2.40)	0.197 (1.74)	(F < 1)
EPz	0.088 (3.44) ${{\rm{\eta }}}_{p}^{2}$: 0.223	(F < 1)	< 0.001* (56.57) ${{\rm{\eta }}}_{p}^{2}$: 0.825	(F < 1)	(F < 1)	0.329 (1.17)	0.156 (2.00)

Statistics are obtained from a univariate repeated-measures ANOVA with 1 degree of freedom (or 2 whenever sign is included as a factor) for the test and 12 degrees of freedom (or 24) for the error. F-values are reported between brackets. Only effects with an F-value larger than 1 are reported in full. Partial eta-squared is reported only for (marginally) significant effects. Significant effects (*p* < 0.05) are marked by*. All abbreviations and conventions as in Table 1.

**Figure 3:**
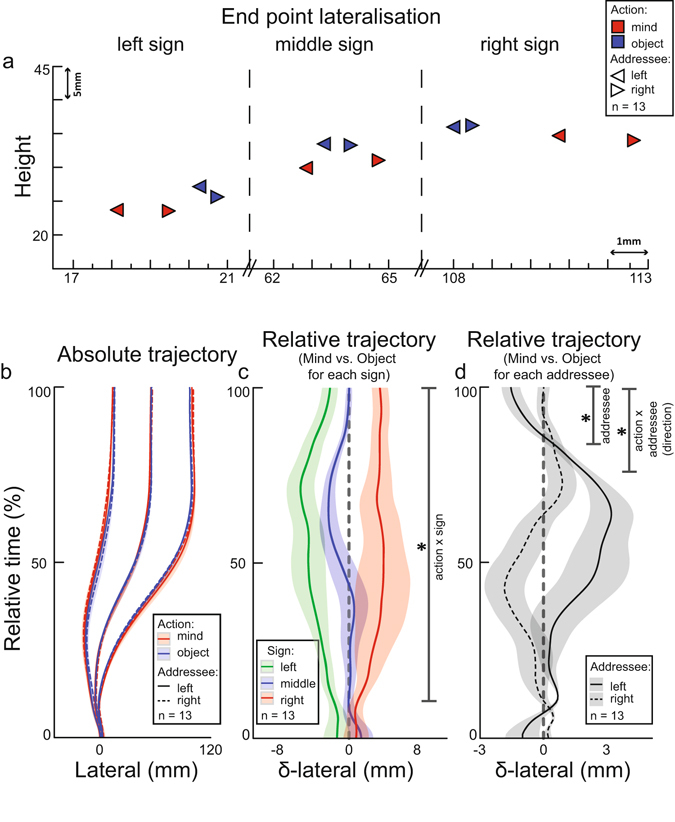
End point locations and lateral development of the trajectory dynamics. (**a**) Pointing end locations towards the left, middle and right signs projected on the horizontal-altitude plane, split for mind- and object oriented actions (red and blue, respectively) and left and right addressees (indicated by arrows). (**b**) Temporal development, relative to the forward movement time, of the horizontal displacement of pointing movements (the ‘lateral development’), split for left, middle and right signs, mind- and object oriented actions (red and blue, respectively) and left and right addressees (continuous lines and dashed lines, respectively). (**c**) The relative difference in the lateral development between mind-oriented and object-oriented pointing movements, split for the left, middle and right signs (green, blue, and red, respectively). *Indicates a significant *action x sign* interaction from 10–100% (*p* < 0.05, corrected). (**d**) The relative difference in the lateral development of mind-oriented and object-oriented pointing movements for the left and right addressee positions. *Indicates a significant *addressee* effect from 83–100% and an *action x addressee* interaction effect in direction (lateral velocity) from 75–99% (both *p* < 0.05, corrected). Variance clouds around the mean trajectories represent standard errors of the mean. The grey dashed lines at point 0 of the relative lateral deviation (in **c** and **d**) represent the baseline object-oriented movements to which the communicative counterparts are contrasted. See the Supplemental Material for the relative lateral deviation split for each combination of sign and addressee.

In addition, we observed differences in the execution of pointing movements towards the different signs (on the backward movement time, the trajectory length, the relative time to peak-velocity, Tables 1 and 2; see the Supplemental Material for further discussion). These main effects of sign show that movements were not symmetrical around the central axis, but converged right of center (Fig. 3b). Importantly, the effects were not specific to any action or addressee condition (*p* > 0.168), suggesting they reflect biomechanical limitations on shoulder-joint rotations when using the right arm to point. Similarly, and probably for the same reason, there was a main effect of sign on the movement time of the addressee (the time in which the addressee selected the correct referent), with both the left and right addressee’s inference being slower when the sign on the left was pointed out (left sign: 1403, middle sign: 1355, right sign: 1322 ms; *F*(2,24) = 17.19, *p* < 0.001, ${{\rm{\eta }}}_{p}^{2}$ = 0.589). Importantly, the addressee’s movement times and error rates were not significantly different between action conditions, an indication that the mind-oriented and object-oriented pointing actions were matched for their consequences on the addressee’s side.

### Pointing end locations

The analysis of the pointing end locations is focused on the horizontal-axis distribution (see the Supplemental Material for a discussion of the depth, altitude and variability of end-points). There were two findings of interest. First, the movement end-points differentiated the three signs more clearly in the mind-oriented than in the object-oriented condition (Fig. 3a): the mind-oriented pointing movements towards the two outer signs fell more outward from the sign (left sign: 18.5 mm, right sign: 111.2 mm) than the corresponding object-oriented movements (left sign: 20.2 mm, right sign: 108.0 mm; *action* x *sign* interaction, *F*(2,24) = 10.29, *p* = 0.001, ${{\rm{\eta }}}_{p}^{2}$ = 0.462). Second, the end-points were shifted towards the location of the addressee: leftward when the communicator interacted with an addressee on the left (63,6 mm, collapsed over signs and actions), and rightward when the communicator interacted with an addressee on the right (64.6 mm; main effect of *addressee*, *F*(2,24) = 14.82, *p* = 0.002, ${{\rm{\eta }}}_{p}^{2}$ = 0.553). Importantly, this effect was driven by the movements produced in the mind-oriented condition (Fig. 3a): the differences between end-point locations when pointing for the left and right addressee were larger in the mind-oriented condition (63.5 and 65.2 mm, for the left and right addressee respectively, collapsed over signs) than in the object oriented condition (63.6 and 64.1 mm; *action* x *addressee* interaction, *F*(1,12) = 6.69, *p* = 0.024, ${{\rm{\eta }}}_{p}^{2}$ = 0.358). These two findings suggest that, during mind-oriented movements, the end-point location of a pointing movement is subtly adjusted to both the location of the addressee and to the spatial layout of potential referents. This indicates that the movement end-point incorporates communicative knowledge specific to the addressee and to the referential context.

### Pointing trajectory dynamics

Similar to the end-point analysis, we focused the analysis of the trajectory dynamics on the lateralization along the horizontal axis, of which the absolute differences can be seen in Fig. 3b (see the Supplemental Material for a discussion of depth dynamics and trajectory variability). There were two findings of interest. First, the trajectories of mind-oriented pointing movements towards the left and right signs were shifted laterally with respect to the corresponding object-oriented pointing trajectories. This ‘mind-oriented lateral shift’ arose early during movement execution, already after 10% of movement duration, and it remained significant until completion of the pointing movement (*p* = 0.003, ${{\rm{\eta }}}_{p}^{2}$ = 0.442; Fig. 3c). Second, the lateral trajectory was also influenced by addressees’ location: whereas movement trajectories seemed to diverge away from the addressee in the initial and middle parts of the movements, at the end they converge in the direction of the addressee: leftward when the communicator interacted with an addressee on the left, and rightward when the communicator interacted with an addressee on the right. This addressee effect was statistically significant from 83% of movement duration onwards (*p* = 0.045, ${{\rm{\eta }}}_{p}^{2}$ = 0.321; Fig. 3d). Analysis of the lateral derivative of the movement (the directional velocity along the x-axis) confirmed that, towards the end of the pointing movements, mind-oriented trajectories were more strongly oriented towards the addressee than the object-oriented trajectories, from 75% until 99% of movement duration (the 100% point is not included due to the deceleration of the end-point; *action* x *addressee* interaction, *p* = 0.009, ${{\rm{\eta }}}_{p}^{2}$ = 0.584; Fig. 3d). This observation shows that, during mind-oriented trials, communicators adjusted the direction of their pointing finger towards the location of the addressee. Together, these findings suggest that, in the presence of stronger communicative demands on the pointing movement itself, the whole pointing trajectory is subtly adjusted to the spatial location of the addressee and to the spatial layout of potential referent objects. This indicates that communicative knowledge specific to the addressee and referential context is incorporated into the trajectory already from an early stage.

## Discussion

This study brings empirical evidence to the ongoing debate on how conceptual knowledge interfaces with sensorimotor processes. We used motion analysis of referential pointing movements to understand the nature of the interface between the conceptual communicative and the sensorimotor systems. We assessed whether communicative knowledge is simply appended to spatially-guided motor routines by means of communicative ostensive cues, or whether that knowledge is also able to access the sensorimotor system and influence the computations that control spatial movement dynamics. There are two main findings. First, the duration of events surrounding the pointing movements, like the time spent holding the hand near the sign, is influenced by the communicative intention, but remains indifferent to the addressee’s point of view. This finding indicates that ostensive cues produced for drawing attention to the communicative status of the action are largely insensitive to the specifics of the triadic relationship between communicator, addressee, and referent. Second, the communicative intention and the location of the addressee influenced the trajectory and the end-point location of the pointing finger. This finding provides evidence for a pervasive interaction between communicative knowledge and sensorimotor computations.

Pointing with the intention to communicate directly to the addressee elicited a longer reaction time (before movement initiation), a prolonged holding time (the finger being held at the end-point) and a longer backward-movement time (from end-point to movement completion). The prolonged reaction time could reflect an increased cognitive load on the communicator. It could also reflect a conversational use of the turn-taking delay, similar to what is observed during spoken dialogue^49^. The prolonged holding time marks and enhances the opportunity for the addressee to accumulate sensory evidence about the spatial relation between the pointing finger and the signs. This interpretation is supported by the observation that the enhanced holding time was most pronounced towards the middle sign, i.e. the sign that was the hardest to distinguish from the others when pointing. These temporal adjustments fit a number of observations obtained across experimentally-controlled communicative interactions as well as naturalistic conversation, in which prolonged timing explicitly marks the communicatively relevant information^21, 50, 51^. Here we add to that literature by showing that these temporal ostensive cues can be adjusted to the estimated difficulty of successfully conveying a message but are not necessarily informed by addressee-specific knowledge.

During mind-oriented pointing, the end location of the index finger marked the relative target locations with a stronger spatial emphasis and varied with the position of the addressee. This shows that the communicator considered the communicative context and the viewpoint of a particular addressee when planning the goal-state configuration of the arm joints in space. Previously, Cleret de Langavant and colleagues^18^ observed a tilted end-point variability when communicative pointing movements were directed towards an addressee on the left (but not on the right) of the communicator. In contrast, we find that movements are systematically and laterally displaced towards the addressee. This discrepancy could be related to differences in procedural details of the two experiments. In the study of Cleret de Langavant and colleagues, communicators ended their movement against a horizontal surface, whereas in this study communicators held their finger in mid-air. Furthermore, in their study, the effects of communicatively pointing towards a single object were compared to those evoked by a non-communicative control condition in which the addressee closed their eyes. In the current study, there were three equiprobable targets, and the effects concern differences between two communicatively relevant conditions. This suggests that the discrepancy between the study by Cleret de Langavant and colleagues and the current study could also be related to different demands in disambiguating the referents.

The lateral shifts revealed in the end-points of communicative trajectories can be interpreted as ostensive cues that are added to an independently programmed movement, or as consequences of the communicative intention directly influencing sensorimotor computations that specify how to reach the desired goal-state. The latter interpretation is supported by the differences in the whole movement trajectory: shortly after movement onset, mind-oriented pointing movements followed a different trajectory than object-oriented movements. Moreover, halfway through the action, communicators adjusted the direction of their movement according to the addressee’s viewpoint. This provides strong support for the idea that mind-oriented actions are planned as a function of the presumed understanding of a particular addressee.

Our findings are in line with previous observations on how instrumental reach-to-grasp movements are affected by characteristics of a particular observer^43–46^. Similarly, this study confirms the observation that lateral movement-shifts, arising late in movement trajectories, are driven by the location of a particular addressee^18^. Crucially, here we show that early effects in movement trajectories are not only affected by the presence of a communicative intention, but also by the array of possible referents. These findings reveal that communicative adjustments are more than exaggerations of an existing motor routine, as suggested by results from the existing literature (e.g. an elevated^18, 30, 45^ or slowly executed movement^21, 22^). We provide empirical evidence, for the first time, for the notion that communicative demands pervasively change a pointing movement, and we show how that movement is influenced by specific features of the triadic relationship between communicator, addressee and object both early and late in action planning.

It could be argued that the communicative relevance of the adjustments observed in the end-points and trajectories of the pointing movements is undermined by the magnitude of those adjustments. Future research should consider whether those adjustments are used by the addressee when inferring a referent. However, the presence of consistent effects on the communicator’s behavior observed here is strong proof of the pervasive and automatic influence that communicative knowledge has on the communicator’s sensorimotor processes. The fact that those effects arise in a situation in which they seem communicatively unnecessary only reinforces this interpretation.

The manipulation of the communicator’s intention that is implemented in this study adds considerable specificity to the empirical findings. Namely, both experimental conditions (mind-oriented and object-oriented movements) required the communicator to share his knowledge with the addressee by means of a pointing movement. The crucial manipulation pertains to the addressee’s requirement to infer the intended reference either directly from the movement or from its instrumental consequence. This subtle manipulation achieves two important effects. First, it minimizes potential confounds arising from coarse between-conditions differences in motivation or social consequences of the actions, intrinsic in previous pointing studies that directly compare communicative with non-communicative settings (e.g. pointing for a blindfolded addressee^18, 22^). Second, this manipulation ensures that the kinematic adjustments do not arise from differences in attentional, perceptual or motoric demands between mind-oriented and object-oriented conditions^52–54^, since these two conditions were identical in terms of their perceptual input, required motor output, and interdependence between the communicator and the addressee.

In conclusion, we studied the interaction between communicative and sensorimotor systems during referential pointing movements and found evidence for a pervasive interaction between those two systems. Pointing trajectories and movement direction were affected by the communicative context and the addressee’s viewpoint, indicating that communicative knowledge has access to low-level sensorimotor computations. This observation opens the way for studying the computational and neural mechanisms that support this pervasive integration between conceptual knowledge and sensorimotor processes.

## Methods

### Participants

Eighteen right-handed participants were recruited (11 female, ages 17–29) and alternated in their role; fifteen played one session as communicator and one session as addressee/onlooker. The other three were assigned the role of addressee/onlooker twice. Two of the fifteen communicators were excluded from analysis because they did not comply with the task requirements (they talked during the experiment and were unable to solve the task consistently: > 30% of trials incorrect). We based our sample size a priori on the sample sizes (9 < n < 14) and observed effect sizes (${{\rm{\eta }}}_{p}^{2}$ ~ 0.5) of previously published studies investigating similar effects^19, 22, 30, 55^. For example, the partial eta-squared of the effect of communicative setting on maximum trajectory deviation observed by Sartori and colleagues^22^ was 0.43, resulting in a testing power (1 − β) > 0.99 with the n = 10 sample size. We used G * Power3^56^ to estimate the required sample size for the current study based on a more conservative prospective effect size: for a repeated measures factorial ANOVA with an effect size of ${{\rm{\eta }}}_{p}^{2}$ = 0.2, a sample size of n = 13 would result in a power (1 − β) > 0.9. The experimental protocol was approved prior to commencement by the local ethics committee (CMO region Arnhem-Nijmegen, The Netherlands). The methods were carried out in accordance with the relevant guidelines and regulations. All participants gave written informed consent and were either given financial compensation or credits towards completing a course requirement.

### Experimental setup

Participants were seated at a table facing two vertically aligned computer screens (Fig. 1a). The top of the bottom screen (56 cm in size) was positioned at eye level of the communicator; the upper screen (same size) was suspended immediately above the bottom screen. Stimuli were visually presented on the two screens, just outside the communicator’s reaching distance, which depended on the participant’s arm length (52 to 57 cm from the participant to the bottom screen). An infrared position detection frame, the ‘IR-frame’ (TMDtouch iMac Zorro Macsk 55 cm, 16:9, USB connected), was used for online measurement of the position of the index finger. The frame spanned a detection plane in front of the bottom screen and within the communicator’s pointing range (13 cm from the home-key, and 17 cm in front of the bottom screen). Communicators sat in front of the frame, with their right index finger resting on a capacitive sensor (the home-key). A chin rest stabilized the head of the communicator and prevented eye contact with the other participants. The addressees/onlookers were oriented towards the center of the screens, left and right at an angle of 40 degrees with respect to the communicator, each with their right hand resting on a keyboard button (the ‘addressee home-key’) and each with a mouse in front of them. At any point in time one of them was engaged in playing the game with the communicator (the addressee), whereas the other was not (the onlooker). A cardboard wall positioned at the top of the bottom screen prevented the eyesight of the addressee on the correct referent for that trial.

### Experimental task

On each trial, three ‘sign’ tokens (colored squares of 4 × 4 cm, Fig. 1) were presented horizontally on the bottom screen for all three participants to see. Three ‘referent’ tokens, also visible to all three participants (Fig. 1a, also shown in the thought cloud of the addressee in Fig. 1b), were presented as two-color circle-triangle composites (4 cm in diameter) on the upper screen. To prevent the use of a communicative strategy based on spatial congruency between the signs and referents, the referents were pseudo-randomly placed on three of nine possible locations of a 3 × 3 grid. At the onset of each trial, only the communicator was visually informed about the correct referent (Fig. 1a, also shown in the communicator’s thought cloud in Fig. 1b). All stimuli were presented against a black background. (Stimulus materials are available at: http://tinyurl.com/OSF-PointingMatters).

The signs could be used to unequivocally identify the correct referent from a set of three through logical rules. Consider the stimuli in Fig. 1b as an example. The communicator is instructed that red-blue is the correct referent, out of the three referents seen by the addressee. He can influence the choice of the addressee by pointing to one of the three signs, i.e. the green, the blue or the red square. Pointing to the blue square would be most informative for the addressee since that sign can be unambiguously associated with the correct red-blue referent, given the colors of the other referents. Pointing to the red square would convey an ambiguous message because that sign can be associated with both the incorrect yellow-red and the correct red-blue referent. Lastly, the green sign can be associated with the incorrect green-magenta referent. These task features, adapted from well-established referential communication games^48^, promote pragmatic reasoning on the mapping between signs and referents. They were introduced to preserve the referential/inferential nature of communicative pointing and minimize participants’ reliance on stereotyped stimulus-response mappings. Accordingly, there was no consistent relationship between the chosen colors of signs and referents, which were randomly selected from a set of six (red, blue, yellow, green, magenta and cyan). Furthermore, in 10% of the trials, only one of the colors in the correct referent appeared in the set of signs. In principle, solving such a trial requires no disambiguation of sign-referent mappings, making the reasoning in the other 90% of the trials less stereotyped.

### Experimental design

The experimental design included three factorial manipulations. First, the factor *action* (two levels: mind-oriented, object-oriented) manipulated how the addressee was provided with information about the referent. In the mind-oriented condition, addressees were dependent on observing the pointing movement of the communicator towards one of the three signs in order to infer the correct referent. The communicator, knowing this dependency, needed to make a pointing movement that the addressee could use to make that inference. In contrast, in the object-oriented condition, the addressee did not need to make that inference, since he was visually informed about the identity of the referent. Second, the factor *addressee* (two levels: left, right) manipulated the role of the other two participants sitting next to the communicator. Across blocks of trials, either the participant on the left or the right of the communicator took the role of addressee with whom the communicator was playing the game (always with the participant on the other side as the onlooker). Third, the factor *sign* (three levels: left, middle, right) varied with the location of the most informative (‘correct’) sign given the referents.

To influence participants through task contingencies rather than through verbal instructions, the game was embedded in a story. The joint goal of the participants was to open a highly secured vault by entering the correct sequence of colored referents. In the object-oriented condition, the computer system of the vault was working properly and the pointing movements were detected by the IR-frame. Hence, when the communicator pointed to a sign and returned to the home-key, the matched referent was displayed on the upper screen for the addressee to see. The addressee simply had to confirm this referent by selecting the identical token from the set of possibilities. Note that the IR-frame was actually used to detect – online – the pointing location and, therefore, the referent shown to the addressee always matched the sign that the communicator pointed to, according to the logical sign-referent mapping described above. This ensured that the pointing action had a direct instrumental consequence, but also that the addressee could be presented with the wrong referent (if the communicator pointed to the wrong sign). In the mind-oriented condition, however, the electronic wiring of the vault was broken. In this condition, the addressee was required to infer the correct referent from the pointing movement of the communicator towards one of the signs.

Note that the story provided a context for the two types of pointing movements. Three aspects of this context are relevant. First, the story emphasized the social nature of the tasks: by engaging in the task, the participants committed themselves to the joint goal of opening the vault together. Second, the story emphasized that the social nature of the task was similar across the two experimental conditions. Third, the story was used to drive the participants towards making inferences on the communicative nature of the pointing movements. Namely, the story nudged the communicators towards the need to inform the addressee about their knowledge in two different ways, without using explicit verbal instructions. These three features of the task are emphasized by the story, but they do not depend on it. Note furthermore that the instrumental consequence of the pointing movement in the object-oriented condition only appeared after completion of the movement, which meant that the perceptual input before movement onset was identical across conditions and could not have driven biases in movement kinematics.

### Experimental procedure

At the start of the experiment, communicators were familiarized with the experimental setup and performed two practice sessions of the game. In the first practice session (8 trials), the participant that would be playing as communicator during the experiment adopted the role of addressee/onlooker. This training was introduced so that the communicator could experience the addressee’s visual perspective and task demands. In the second practice session (8 trials), the participant adopted the role of communicator. There were no constraints on the pointing movements, other than to move the index finger towards the screen, going through the 27.5 × 48.5 cm IR-frame, and return to the home-key.

The experiment consisted of three sessions of twenty minutes. There were 240 trials in total, grouped in blocks of ten trials with a short break in between. The correct-referent (and thereby the corresponding sign) was pseudo-randomly determined for each trial. The roles of addressee and onlooker changed every block. The mind-oriented and object-oriented actions changed every two blocks. Before each block, participants were informed of the forthcoming conditions, i.e. where the addressee/onlooker was located, and whether or not the vault-system was going to inform the addressee about the referent.

During a given trial all participants were required to have their fingers on their home-keys. At the start of a mind-oriented trial, the addressee (not the onlooker) triggered the presentation of signs and referents by pressing the space bar on their keyboard. In the object-oriented trials, similar inter-trial intervals were introduced by having a computer presenting the stimuli after a random time interval (between 1.4 and 3.4 seconds). Once the communicator returned to the home-key after the pointing movement, a cursor appeared in between the referents on the upper screen. This allowed the addressee (not the onlooker) to control the cursor with a mouse, select one of the referents with a mouse-click and return to their home-key. Trial-by-trial feedback was given in the form of a thick red (incorrect) or green (correct) line surrounding the stimuli. Aggregate feedback on performance was provided at the end of each block (“Congratulations, you have successfully opened the vault” when all ten trials were correct; “Sorry, you were not successful in opening the vault” otherwise).

After completing all trials the participants were debriefed. Participants indicated that they were focused on solving the task successfully and they felt that their behavior was important for achieving that goal. Most of the communicators did not experience any differences in complexity between the mind-oriented and object-oriented pointing conditions while a couple of them indicated the mind-oriented condition to be more difficult because the addressee depended on seeing their movement. In line with this, only a few participants indicated that they explicitly considered the perspective of the addressee. Although it was obvious to all participants that their arm movements were being recorded, they did not know which features of those movements were monitored or what the experimental expectations of them were.

### Data-acquisition and analysis

The position and orientation of four sensors placed on the communicator’s right hand was sampled at 250 Hz (and 0.1 mm resolution) using an electromagnetic tracking system (LIBERTY, Polhemus). One sensor was placed on the distal phalanx of the index finger of the right hand just below the nail (index). Two sensors were placed on the hand, one on top of the second metacarpophalangeal joint (hand-radial) and the other on top of the fifth metacarpophalangeal joint (hand-ulnar). One sensor was positioned on the distal side of the corpus radii (wrist). As there were comparable results across the four sensors, we focused on the data obtained from the index-finger sensor. (Data is available at http://tinyurl.com/OSF-PointingMatters and tools for the analyses are available at https://github.com/lennartverhagen/kinemagic).

Kinematic data were analyzed using MATLAB (MathWorks, Natick, MA, USA). A detailed specification of the data processing and analysis procedures can be found in^57^; here we provide a summary description. The time-series of positions/orientations of each sensor were low-pass filtered at 15 Hz using a sixth-order Butterworth filter.

### Task phases

We considered the following task phases. The *reaction time communicator* was defined as the time from stimuli presentation to initiation of the movement (marked by the release of the home-key). The *movement time addressee* was defined as the time from the release of the addressee’s home-key to selection of one of the referents. Within the movement phase of the communicator, several subcomponents were distinguished. We took particular care to robustly estimate their onsets and offsets by combining both distance and velocity parameters^58^. The *forward movement time* was defined as the time where the velocity of the index finger in the sagittal (yz) plane had increased and remained above 0.1 m/s while moving from the home-key to the end-point holding position. The *holding time* was defined as the time the end-point holding position was maintained, i.e. where the index finger remained at a velocity below 0.1 m/s, while in proximity of the signs. Lastly, the *backward movement time* was defined as the time where the velocity of the index finger in the sagittal (yz) plane had increased and remained again above 0.1 m/s, while moving from the end-point holding position back to the home-key. We ensured that all forward movements started in proximity of the home-key and ended in proximity of the signs. We included only movements that started no more than 10 cm above and 8 cm in front of the home-key and ended no more than 10 cm above, no more than 15 cm below and no more than 15 cm to the left or right of the signs. We used the same spatial restrictions to ensure that all backward movements started in proximity of the signs and ended in proximity of the home-key.

### Kinematic parameters

We describe the pointing movements using the following kinematic parameters: *trajectory length*, *peak velocity*, and *relative time-to-peak velocity* (as a fraction of the forward movement time)^59^. Furthermore, we extracted and aggregated the spatio-temporal dynamics of the pointing trajectories as follows. First, for each trial, 100 samples along the forward movement trajectory were isolated at equally spaced time intervals using local spline interpolation. For each sample, we extracted the position along the x, y and z axis and calculated a signed numerical instantaneous derivative to describe the *horizontal displacement* (lateral, x-axis), *depth* (forward, y-axis) and *altitude* (height, z-axis). The spatial trajectory of the pointing movements was also used to quantify the *end-point* (in x, y and z) and *trajectory variability*. Additionally, we described the spatial movement variability as a function of progression along the trajectory. Aggregating trajectories, while ignoring temporal biases, is a non-trivial operation that involves iterative optimization^60, 61^. Our approach considered 300 equidistant samples along the spatially averaged trajectory. At each sample we calculated the linear intersection of all individual trajectories with a plane perpendicular to the average instantaneous movement direction at that point in space. This resulted in series of 300 2D distributions of points (oriented in 3D). For each distribution the confidence ellipse was calculated on the basis of an Eigen-decomposition of the covariance matrix of this set of points and scaled using a *χ* ^*2*^ distribution to match a 95% confidence interval in 2D^62^. The critical parameter describing the variability of the trajectories along the movement is the area of the confidence ellipses.

### Statistical inference

We excluded the first two trials of the first block (0.8%) because we considered those to be prone to learning effects. Participants communicated effectively: on average 92.4% of the trials were solved correctly by both players. The remaining 7.6% of the trials were excluded from the analysis (the communicator pointed to a wrong sign, 1.9%, the addressee selected a wrong referent, 2.7%, or both participants selected the wrong token, 3.0%). Finally, we excluded trials where the reaction time of the communicator exceeded 8 seconds (0.6%) and outlier trials where any of the main kinematic parameters deviated from the first or third quartile by more than three interquartile ranges (4.7%). We also rejected trials where no valid movement was detected (2.1%), either because no movement was made, or the movement started away from the home-key (>10 cm above or >8 cm in front of the home-key), or the detected holding position was not in proximity of the signs (>10 above, >15 cm below, or >15 cm to the left or right of the signs). In total, 84.1% of all trials (per action: for mind-oriented 81.6% and object-oriented 86.4%, per addressee: left 84.6% and right 83.5%, per sign: left 80.3%, middle 85.2% and right 86.5%) survived the exclusion criteria and entered further analysis.

Statistical inference (of task phases, movement parameters and end-points) was drawn using the SPSS 16.0 software package. Trials were averaged for each experimental condition, and the resulting means were entered into a univariate repeated-measures ANOVA testing for main and interaction effects between conditions within subjects. All parameters were normally distributed (Kolmogorov-Smirnov test). The statistical inference of the spatio-temporal dynamics is not possible by means of similar univariate or multivariate approaches, because of the inherent dependencies between data points neighboring in space and time. Therefore, we chose to use non-parametric cluster-based permutation statistics^63^. We obtained an accurate Monte Carlo estimate of the true *p-*value (corrected for multiple comparisons) by comparing the cluster statistics of interest to the distribution of cluster statistics calculated from 10,000 random permutations of the conditions (using a critical alpha-level of 0.05). With exception of the permutation statistics, all statistical tests were performed two-sided.

## Electronic supplementary material

Supplementary Information 
