# Supplementary material for: Communicative knowledge pervasively influences sensorimotor computations
Source: Sci Rep. 2017 Jun 27;7:4268. doi: 10.1038/s41598-017-04442-w (PMC5487354; doi:10.1038/s41598-017-04442-w)
Supplement: Supplementary file 1 — Supplementary Information [file 41598_2017_4442_MOESM1_ESM.doc]

# **­­­Supplemental material**

**Communicative knowledge pervasively influences sensorimotor computations.**

Anke Murillo Oosterwijk^1,2^*, Miriam de Boer^1^, Arjen Stolk^3^, Frank Hartmann^2^, Ivan Toni^1^, Lennart Verhagen^4^

Radboud University Nijmegen, Donders Institute for Brain, Cognition and Behaviour, The Netherlands. E-mail: a.murillooosterwijk@donders.ru.nl

^2^Erasmus University Rotterdam, Erasmus Research Institute of Management, Accounting, The Netherlands

^3^Helen Wills Neuroscience Institute, University of California, Berkeley, USA

^4^University of Oxford, Department of Experimental Psychology, United Kingdom

**Effects of sign on task phases, movement parameters and end-points**

First, the backward movement time was slower (*F*(2,24) = 14.21, *p* < .001, η*_p_*^2^ = .809; Table 2) and the peak velocity was reached at a later point in time for signs positioned further on the right (relative peak velocity: *F*(2,24) = 10.02, *p* = .001, η*_p_*^2^ = .455). Not surprisingly, in both mind-oriented and object-oriented pointing movements, the finger converged in the direction of the intended referent sign (EPx: *F*(2,24) = 1629.66, *p* <.001, η*_p_*^2^ = .993). Second, the trajectory of the communicator's index finger was longer for movements towards signs positioned further on the right independent of action type (*F*(2,22) = 99.14, *p* < .001; η*_p_*^2^ = .892). This is in line with the end point locations of the middle sign being further away and higher than the left one, and the right one being further away and higher than the middle one (EPy: *F*(1,24) = 237.49, *p* < .001, η*_p_*^2^ = .952; EPz: *F*(1,24) = 56.57, *p* < .001, η*_p_*^2^ = .825). These main effects of sign likely reflect the fact that the communicator used the right arm to point and the concomitant limitations on shoulder-joint rotation.

**Effects on forward and development of trajectories**

We observed a main effect of action and of addressee on the forward development of pointing movements (Fig. S1). First, pointing movements in the mind-oriented condition developed more forward than those in the object-oriented condition from 52% of movement duration onward (*p* = .004). Second, trajectories for the right addressee developed more forward than those for the left addressee, but only in the communicative condition, from 33% until 53% of the movements (*p* = .049). Congruently, the peak-velocity of the forward movement was reached relatively later when pointing for the right addressee than for the left addressee (*F*(1,12) = 9.98, *p* =.008, η*_p_*^2^ = .454), irrespective of the presence of communicative intent.


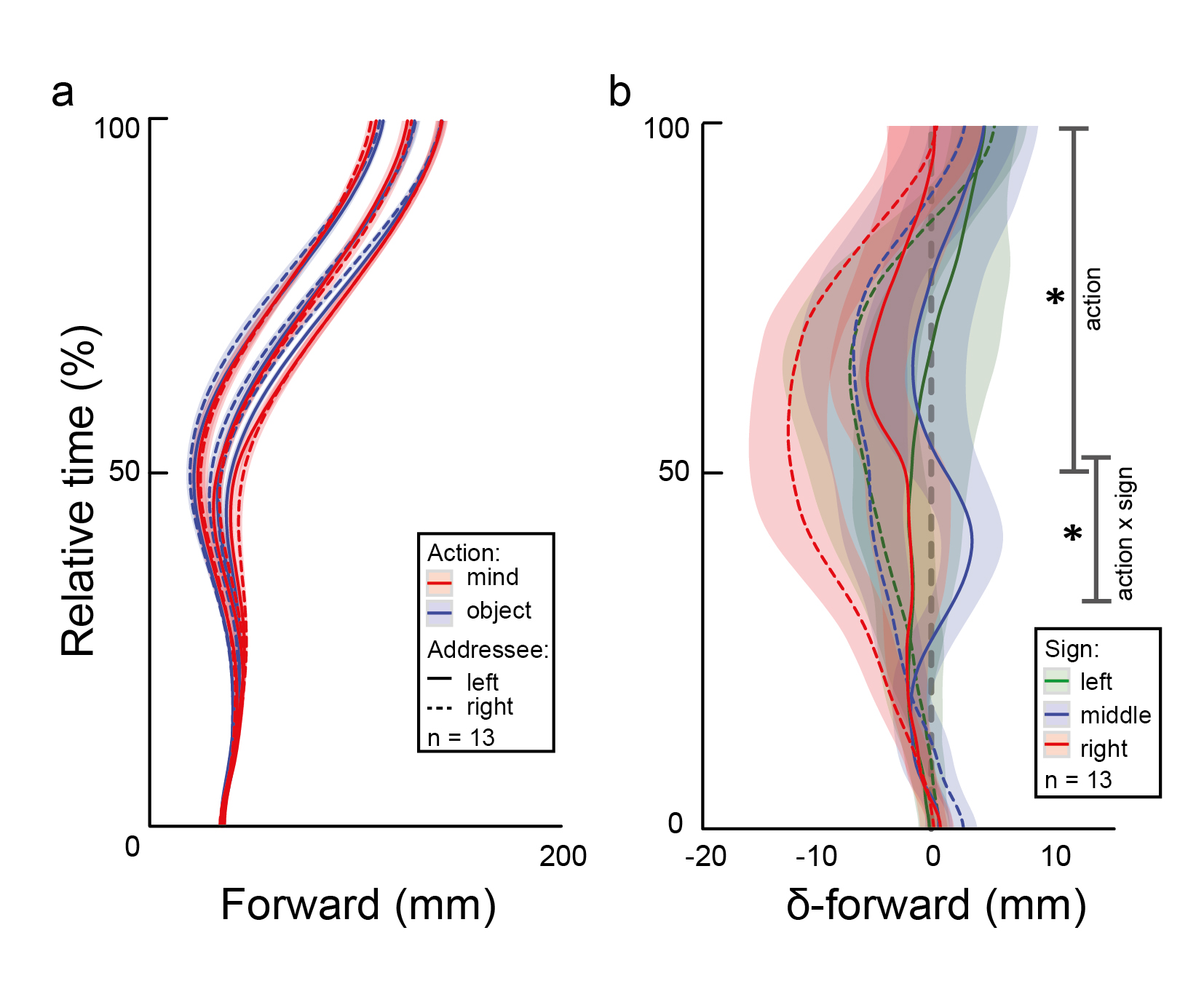


**Figure S1: Forward development of the temporal trajectory dynamics**. a. Spatio-temporal development, relative to the forward movement time, of the forward displacement of pointing movements, split for mind- and object oriented actions (red and blue, respectively) and left and right addressee positions (continuous lines and dashed lines, respectively). b. The relative difference in forward development between mind-oriented and object-oriented pointing movements for the left, middle and right signs (green, blue and red, respectively) and the left and right addressee positions (continuous lines and dashed lines, respectively). * indicates a significant *action* effect from 52-100% and a significant *action x sign* interaction from 33-53% (both p < .05, corrected).

**Effects on forward and development of trajectories**

As a supplement to the effects already shown and discussed (see Figure 3c and 3d), here we show the relative trajectories split for all combinations of signs and addressees (Fig. S2).

**
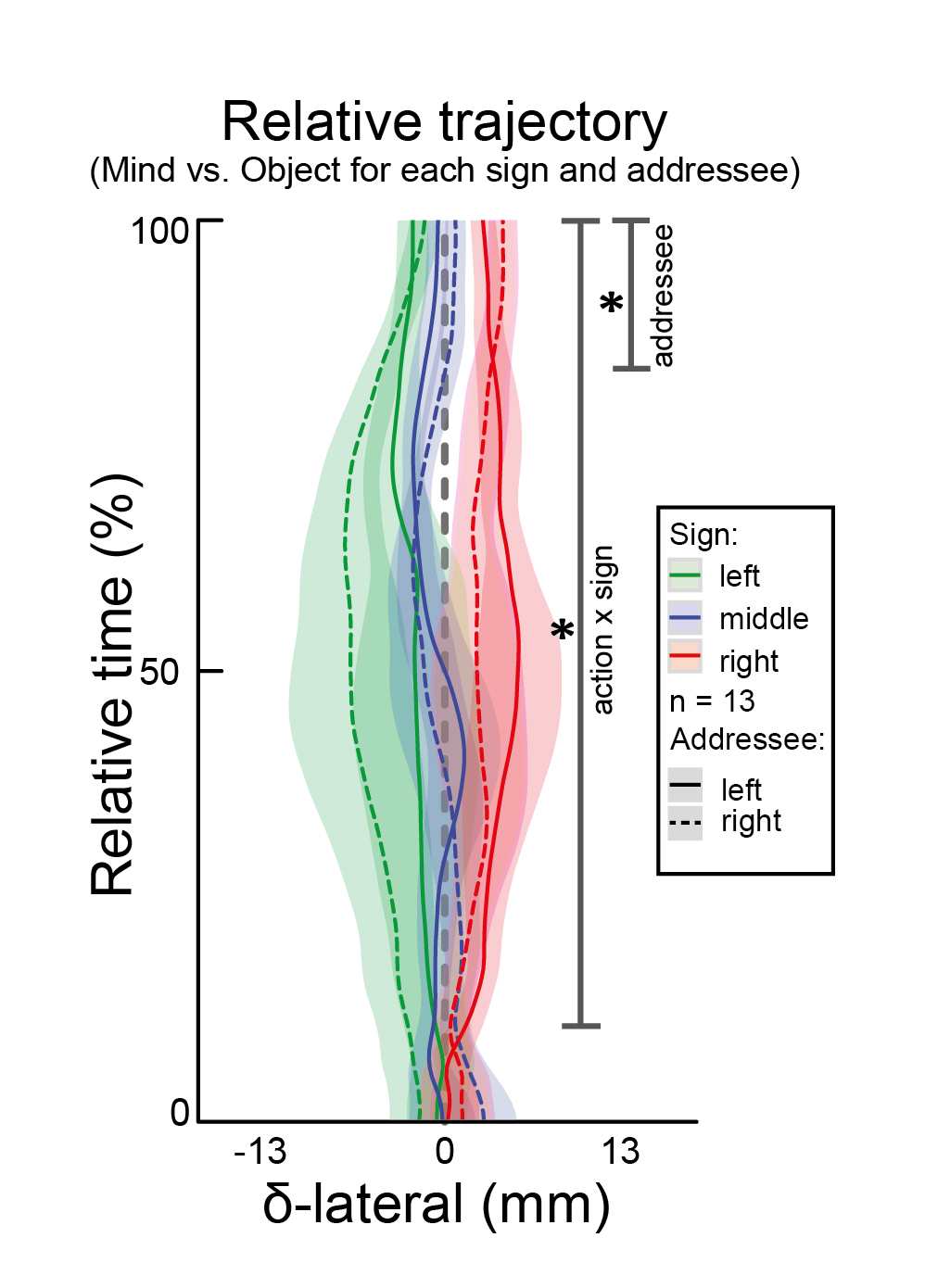
**

**Figure S2: Lateral development of the trajectory dynamics.** The relative difference in the lateral development between mind-oriented and object-oriented pointing movements, split for the left, middle and right signs and left and right addressee positions. * indicates a significant *action x sign* interaction from 10-100% and a significant *addressee* effect from 83-100% (*p* < .05, corrected).

**Effects in spatial variability of trajectories**

Lastly, the within-subject spatial variability of the pointing trajectories was higher for mind-oriented than object-oriented pointing, surviving cluster-level significance at two phases of the movement, between 43% and 61% (*p* = .006, η*_p_*^2^ = .195) and 67% and 100% of movement duration (*p* = .044, η*_p_*^2^ = .429; Fig. S3a). No systematic differences were found in the end-point distribution as can be seen in Figure S3b.

**
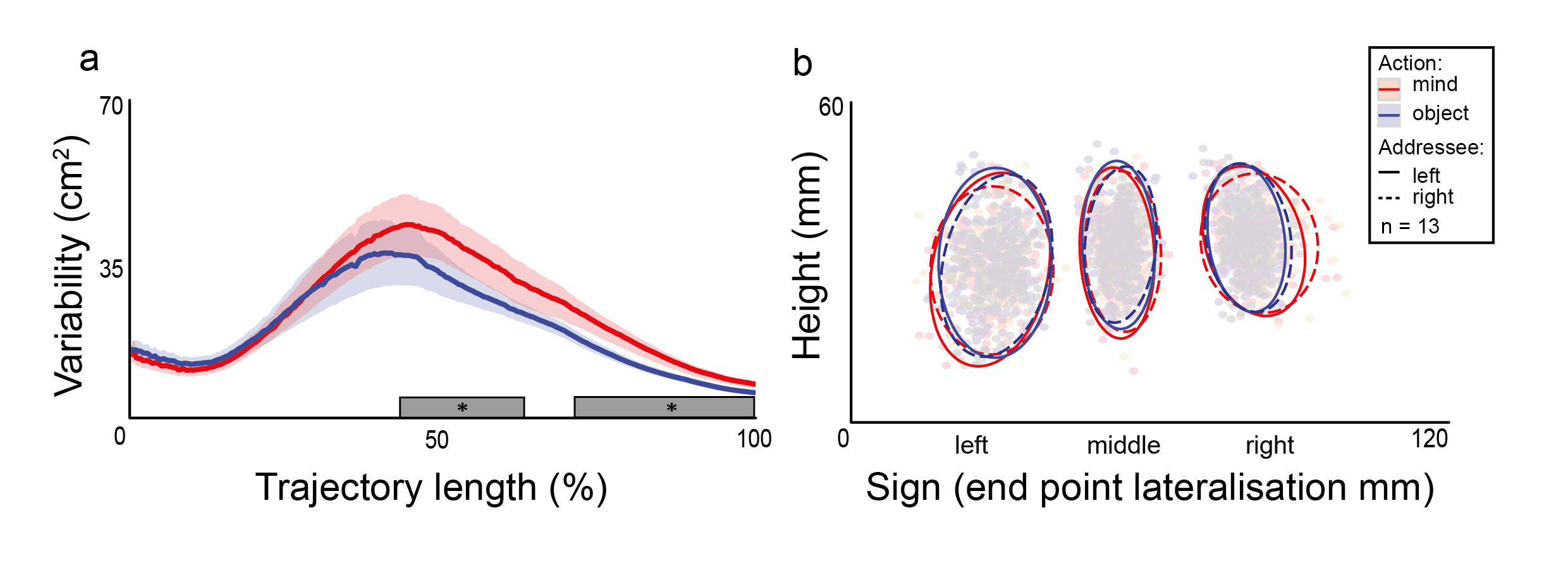
­**

**Figure S3: Trajectory and end-point variability.** a. The area of confidence ellipse during the trajectory of mind and object oriented pointing movements. Variance clouds around the mean represent standard errors of the mean. * indicates a significant difference at 43-61% and 67-100% (both p < .05, corrected). b. Individual end points and confidence ellipses of pointing movements towards the left, middle and right signs, split for mind- and object oriented actions (red and blue, respectively), left and right addressees (continuous lines and dashed lines, respectively).

| **Factor** | | | | **Action** | | | | | | **Addressee** | | | | | | **Sign** | | | | | | | | |
| --- | --- | --- | --- | --- | --- | --- | --- | --- | --- | --- | --- | --- | --- | --- | --- | --- | --- | --- | --- | --- | --- | --- | --- | --- |
| **Condition** | | | | Mind | | | Object | | | Left | | | Right | | | Left | | | Middle | | | Right | | |
| **RTc** | | *ms* | | 2043 (43) | | | 1945 (39) | | | 2012 (42) | | | 1977 (40) | | | 2041 (50) | | | 1977 (48) | | | 1964 (54) | | |
| **MTf** | | *ms* | | 885 (12) | | | 856 (15) | | | 877 (15) | | | 864 (13) | | | 876 (18) | | | 873 (17) | | | 863 (18) | | |
| **HT** | | *ms* | | 426 (42) | | | 225 (30) | | | 327 (38) | | | 323 (39) | | | 301 (45) | | | 343 (49) | | | 332 (46) | | |
| **MTb** | | *ms* | | 749 (13) | | | 704 (12) | | | 732 (14) | | | 722 (12) | | | 738 (15) | | | 729 (16) | | | 713 (16) | | |
| **MTa** | | *ms* | | 1359 (27) | | | 1361 (27) | | | 1396 (22) | | | 1324 (30) | | | 1403 (32) | | | 1355 (32) | | | 1322 (33) | | |
| **TL** | | *cm* | | 41.7 (0.6) | | | 42.1 (0.6) | | | 42.0 (0.6) | | | 41.9 (0.5) | | | 39.7 (0.7) | | | 41.6 (0.6) | | | 44.4 (0.6) | | |
| **PV** | | *cm/s* | | 101.8 (2.2) | | | 104.2 (2.6) | | | 103.2 (2.3) | | | 102.8 (2.5) | | | 101.6 (3.3) | | | 103.0 (2.9) | | | 104.3 (2.6) | | |
| **rtPV** | | *%* | | 16.4 (0.9) | | | 17.7 (1.0) | | | 16.5 (0.9) | | | 17.6 (0.9) | | | 16.6 (1.2) | | | 15.2 (1.0) | | | 19.4 (1.1) | | |
| **EPx** | | *mm* | | 64.4 (4.4) | | | 63.8 (4.1) | | | 63.6 (4.2) | | | 64.6 (4.2) | | | 19.3 (0.9) | | | 63.4 (0.5) | | | 109.6 (0.6) | | |
| **EPy** | | *mm* | | 33.6 (3.2) | | | 36.1 (3.2) | | | 35.1 (3.3) | | | 34.6 (3.1) | | | 16.2 (3.5) | | | 35.8 (3.4) | | | 52.6 (3.2) | | |
| **EPz** | | *mm* | | 29.9 (1.3) | | | 32.6 (1.4) | | | 31.4 (1.4) | | | 31.1 (1.3) | | | 24.9 (1.7) | | | 32.7 (1.5) | | | 36.2 (1.3) | | |
|  | **Action x Addressee** | | | | | | | **Action x Sign** | | | | | | | | | **Addressee x Sign** | | | | | | | |
|  | Mind | | | | Object | | | Mind | | | | Object | | | | | L. A. | | | | R. A. | | | |
|  | L. A. | | R. A. | | L. A. | R. A. | | L. S. | M. S. | | R. S. | L. S. | | M. S. | R. S. | | L. S. | M. S. | | R. S. | L. S. | | M. S. | R. S. |
| **RTc** | 2061 (61) | | 2025 (61) | | 1962 (58) | 1928 (52) | | 2050 (77) | 2042 (69) | | 2038 (80) | 2033 (66) | | 1912 (65) | 1891 (70) | | 2067 (74) | 1981 (70) | | 1988 (78) | 2016 (70) | | 1973 (66) | 1940 (75) |
| **MTf** | 898 (20) | | 872 (15) | | 856 (22) | 856 (22) | | 891 (23) | 885 (22) | | 878 (21) | 860 (28) | | 860 (25) | 848 (28) | | 882  (28) | 888  (24) | | 860  (26) | 869  (23) | | 857  (23) | 865  (24) |
| **HT** | 412 (57) | | 440 (62) | | 243 (45) | 206 (40) | | 387 (70) | 460 (78) | | 432 (72) | 216 (51) | | 227 (54) | 231 (53) | | 293 (61) | 354 (72) | | 335 (64) | 309 (66) | | 333 (69) | 328 (69) |
| **RTb** | 751 (20) | | 748 (17) | | 712 (18) | 696 (16) | | 760 (22) | 757 (23) | | 732 (23) | 716 (20) | | 702 (21) | 694 (22) | | 739 (24) | 737 (22) | | 718 (25) | 737 (19) | | 722 (23) | 707 (21) |
| **MTa** | 1385 (28) | | 1334 (45) | | 1408 (35) | 1314 (40) | | 1398 (43) | 1379 (48) | | 1301 (47) | 1409 (49) | | 1331 (43) | 1343 (49) | | 1431 (37) | 1394 (41) | | 1364 (38) | 1375 (53) | | 1316 (49) | 1280 (55) |
| **TL** | 41.9 (0.8) | | 41.6 (0.8) | | 42.0 (0.8) | 42.1 (0.7) | | 39.5 (1.0) | 41.5 (0.9) | | 44.3 (0.9) | 40.00 (0.9) | | 41.8 (0.8) | 44.5 (1.0) | | 39.9  (0.9) | 41.7 (0.9) | | 44.3 (1.0) | 39.6 (0.9) | | 41.5 (0.8) | 44.5  (0.8) |
| **PV** | 101.8  (2.9) | | 101.8  (3.3) | | 104.6  (3.6) | 103.7  (3.8) | | 100.4 (4.7) | 102.1  (3.5) | | 102.8 (3.1) | 102.8 (4.6) | | 103.9 (4.8) | 105.8 (4.3) | | 101.2 (4.2) | 103.3 (4.1) | | 104.9 (3.9) | 101.9 (5.0) | | 102.7 (4.3) | 103.7 (3.6) |
| **Rt**  **PV** | 15.9 (1.2) | | 17.0 (1.2) | | 17.0 (1.4) | 18.3 (1.5) | | 16.4 (1.6) | 14.6 (1.3) | | 18.3 (1.4) | 16.8 (1.9) | | 15.8 (1.6) | 20.4 (1.6) | | 16.8 (1.8) | 14.2 (1.4) | | 18.3 (1.5) | 16.3 (1.8) | | 16.2 (1.5) | 20.4 (1.6) |
| **EPx** | 63.5 (6.2) | | 65.2 (6.2) | | 63.6 (5.9) | 64.1 (5.9) | | 18.5 (1.3) | 63.4 (0.7) | | 111.2  (1.0) | 20.2 (1.2) | | 63.3 (0.6) | 108.0  (0.6) | | 18.9 (1.2) | 62.8 (0.7) | | 109.1  (0.8) | 19.7 (1.3) | | 64.0 (0.6) | 110.1 (1.0) |
| **EPy** | 33.8 (4.6) | | 33.4 (4.5) | | 36.4 (4.7) | 35.8  (4.4) | | 13.9 (4.7) | 34.3 (4.9) | | 52.6 (4.5) | 18.4 (5.1) | | 37.4 (4.9) | 52.5 (4.8) | | 17.4  (5.0) | 35.1 (5.3) | | 52.8 (4.8) | 15.0 (4.8) | | 36.6 (4.5) | 52.3 (4.5) |
| **EPz** | 29.9 (1.9) | | 29.9 (1.7) | | 33.0 (1.9) | 32.3 (2.0) | | 23.4 (2.3) | 31.1 (2.0) | | 35.2 (1.8) | 26.4 (2.5) | | 34.3  (2.1) | 37.3 (2.0) | | 25.4 (2.5) | 32.5 (2.2) | | 36.4  (1.9) | 24.5 (2.3) | | 32.9 (2.0) | 36.0 (1.9) |

**Table S1: Descriptive statistics: mean and standard error of the mean per action, addressee and sign effects.** All abbreviations and conventions as in Table 1.
